# Medical Examiners for the State of Georgia

**Published:** 1895-01

**Authors:** J. McF. Gaston


### MEDICAL EXAMINERS FOR THE STATE OF GEORGIA.

The bill which passed the legislature of Georgia for the regulation of the practice of medicine in the State, permits all who were entitled at the date it went into effect to exercise the office of physician or surgeon, to continue in the discharge of their duties.

The large brood of quacks developed in the city of Atlanta, by the laxity of our laws heretofore, will doubtless presume upon this recognition to assert their claims to a place in the medical profession, from which they have been debarred by ethical standards. It, therefore, devolves upon the practitioners of good standing in the regular school, to draw the lines now more distinctly than ever, to protect the medical profession from the encroachments of those not conforming to the rules and regulations of the ethical code.

This applies not only to those holding diplomas from schools not recognized as regular, but to the graduates of regular schools who are not following the lines of practice inculcated by their schools and by the Medical Association of Georgia under the code of ethics laid down for the guidance of the profession by the American Medical Association. There are now in this city and in other cities of Georgia, a large number of regular graduates who are engaged in the practice of quackery in its various forms, and so far as the enactment of the bill goes, they are at liberty to keep up those practices. But they are not entitled to recognition by the regular practitioner in pursuing this devious course and should be ruled out of the medical profession. Those who are openly resorting to a species of advertisement not consistent with medical ethics, should be placed by all medical men of good repute in the category of quacks, who are debarred from recognition by consultation or otherwise. If these members of the medical profession prefer such irregular procedures to a compliance with the code of ethics, we can only say that "Ephraim is joined to his idols, let him alone." But those who prize their good name in the profession should wipe their hands from the stain of professional association with all such degenerate sons of Æsculapius.

The working of the new law will effectually rule out all such vultures as Flower, who has been preying upon the people of Atlanta and other southern cities, during the past few years.

The traveling firms of so-called doctors who have spent some weeks or months of each year in our city, have imposed upon the credulity of those suffering from imaginary ailments and have reaped a large harvest of pecuniary gain from people who were little able to meet their exactions and who received in most cases no benefit whatever from their treatment.

The authorities should keep a close watch upon these harpies in future and compel them to move on when disposed to stop here.

In respect to the bearing of the bill upon the medical colleges and the graduates of the same for the future, it remains to be seen what will be accomplished for the welfare of the community, in supplying competent physicians.

The requirement of a three years' graded course of lectures of six months each must necessarily afford better preparation for the practice of medicine and surgery than the former two years' course of five months each, with a simple repetition of the same lectures.

What is still of vital importance remains undone by this bill, and is not sufficiently insisted upon by any of the medical schools, that is, the preliminary training of the student by a competent preceptor before entering upon a course of medical lectures. Fifty years ago this requirement was fulfilled in the case of most students before going to a medical college, and when a young man went into the lecture-room with this preparation, he could take in more satisfactorily what was taught by the professors in the various departments.

This omission is not met by the requirement of the Southern Medical College Association for preparatory scholarship in the ordinary branches of knowledge before entering upon the study of medicine. However necessary this may be to properly comprehend the instruction of the lecture-room, it cannot supply the deficiency of a special preceptor in the rudimentary departments of anatomy, physiology, materia medica and chemistry for the medical student.

Unfortunately, a large proportion of the young men who have entered our medical colleges in recent years, have not possessed the ordinary schooling or the elementary medical training which should prepare them for a course of lectures in a medical college.

The natural consequence of this lack of preparatory instruction is manifested by their inability to comprehend what is taught by the professors and with the multiplicity of matters presented each day, they become confused and receive little profit.

This precipitation will be very materially modified by the operation of the medical examining board, which must tend to impress upon prospective students the importance of proper preparatory study.

The division of the medical examiners into three boards of five members each, which meets the diversity of teaching in the regular, eclectic and homœpathic school, ought to be satisfactory to all parties. If the men who have been appointed by the Governor to constitute the regular board of examiners do their duty faithfully and discreetly, no graduate of any regular school of medicine in Georgia who is fitted to enter upon the duties of his profession ought to be rejected.

If from any cause a young man gets hold of a diploma without proper qualification, this examination by the board is intended to put a check upon his wild career of adventure and by reminding him that he needs further instruction, a great benefit will be conferred upon him, and a greater still upon the community in which he expects to practice.

It is to be expected that personal influence may seek to gain favor for candidates at times, and yet the members of the board should prove themselves independent of all such favoritism.

The wise provision that no member of the medical board shall be connected with any of the medical colleges should prove an effectual guarantee against any partiality for the students of either school.

The competition of the different medical schools in Georgia will in future resolve itself into a decision in favor of that one which affords the best training for the duties of the physician and surgeon. A student will no longer raise the question as to the school in which he can get a diploma easiest and with the least outlay. His inquiry will be directed to getting assurance of the attainment of that knowledge of the different branches which will enable him to pass a satisfactory examination by the faculty for graduation, and subsequently, to meet the requirements of the board of medical examiners for the State of Georgia, who are sworn to do their duty fairly and justly. So mote it be.

J. McF. G.

